# Heterogeneity in longitudinal medication adherence patterns among patients with spinal tuberculosis: a latent class analysis using multi-timepoint follow-up data and associations with clinical outcomes

**DOI:** 10.3389/fpubh.2026.1733775

**Published:** 2026-03-12

**Authors:** Na Wang, Haijing Xiao, Yujuan Liu, Yawen Ma, Liping Wu, Qianqian Wang, Zheng Wang, Jine Chen, Xi Zhang

**Affiliations:** 1 The Second Department of Orthopaedic Trauma, General Hospital of Ningxia Medical University, Yinchuan, China; 2 Outpatient Department of the People's Hospital of Ningxia Hui Autonomous Region, People's Hospital of Ningxia Hui Autonomous Region, Yinchuan, China; 3 Department of Outpatient Services, General Hospital of Ningxia Medical University, Yinchuan, China; 4 Department of Hepatobiliary Surgery, General Hospital of Ningxia Medical University, Yinchuan, China; 5 Department of Nursing, Cardio-Cerebrovascular Hospital, General Hospital of Ningxia Medical University, Yinchuan, China

**Keywords:** dynamic trajectory, group-based trajectory model, influencing factors, medication adherence, spinal tuberculosis

## Abstract

**Objective:**

This study aims to explore the dynamic trajectory of medication adherence behavior and its influencing factors among patients with spinal tuberculosis within 6 months after discharge, providing a basis for developing individualized intervention strategies.

**Methods:**

A retrospective analysis was conducted using data from a longitudinal follow-up cohort, enrolling 117 spinal tuberculosis patients who underwent surgical treatment at the General Hospital of Ningxia Medical University between January 2024 and August 2025. Data were collected via telephone follow-up at 1 week, 1 month, 3 months, and 6 months after discharge, including general demographic information, scores from the Morisky Medication Adherence Questionnaire, Oswestry Disability Index (ODI), and laboratory indicators such as erythrocyte sedimentation rate and C-reactive protein levels. Group-based trajectory modeling (GBTM) was employed to identify subgroups of medication adherence trajectories, and multivariate logistic regression analysis was conducted to examine the influencing factors of these trajectory patterns.

**Results:**

Among 117 patients, group-based trajectory modeling identified three medication adherence trajectories: “persistently high adherence” (35.0%, *n* = 41), “fluctuating decline” (48.7%, *n* = 57), and “early low adherence” (16.2%, *n* = 19). The early low adherence group was older (64.21 ± 5.92 years) and had higher baseline ODI (62.11 ± 5.15) and CRP (39.58 ± 12.40 mg/L). Factors associated with non-adherence in the fluctuating vs. high adherence group included higher ODI *z*-score (OR = 4.658, 95% CI: 3.851–5.581), adverse drug reactions (OR = 3.249, 95% CI: 2.664–3.868), older age *z*-score (OR = 1.974, 95% CI: 1.524–2.516), and higher log(CRP+1) (OR = 1.532, 95% CI: 1.314–1.776). Protective factors were family supervision (OR = 0.779, 95% CI: 0.633–0.943), higher education (OR = 0.576, 95% CI: 0.498–0.660), and higher monthly income *z*-score (OR = 0.471, 95% CI: 0.373–0.583). All analyses were based on 1,000 bootstrap samples with L2 regularization.

**Conclusions:**

Medication adherence behavior among spinal tuberculosis patients exhibits heterogeneous dynamic trajectories. Clinically, tailored interventions should be developed according to the characteristics and influencing factors of different trajectory subgroups, with particular attention to older patients, those with lower educational levels, those experiencing adverse drug reactions, and those lacking supervision, in order to improve medication adherence.

## 1 Introduction

Spinal tuberculosis, being the most common type of bone and joint tuberculosis (accounting for over 50%), is characterized by a long and debilitating course with far-reaching consequences (1–3). The invasion of the spine by *Mycobacterium tuberculosis* gradually destroys the vertebral structure, leading to serious complications such as spinal deformities and neurological dysfunction (4–6). In severe cases, it can even result in paralysis, significantly reducing patients' quality of life. Anti-tuberculosis drug therapy remains the cornerstone of treatment for this condition and must strictly follow the principles of “early initiation, combination therapy, appropriate dosage, regular administration, and full course.” For newly diagnosed patients, the treatment duration is at least 6 months, while drug-resistant patients require even longer treatment periods (7–9). However, in clinical practice, patient adherence to medication regimens is generally poor. Research by Louw has shown that only 21.33% of patients with spinal tuberculosis maintain good medication adherence (10). Poor adherence has serious consequences, not only increasing the recurrence rate of the disease to as high as 50%, but also significantly raising the risk of drug resistance, creating a vicious cycle that is difficult to break (11).

Current research on medication adherence among patients with spinal tuberculosis mostly relies on cross-sectional surveys (12, 13). Such studies can only reflect patients' adherence status at a single point in time and are unable to reveal the dynamic changes throughout the entire treatment process. As a result, they fail to capture potential behavioral fluctuations at different stages—for example, patients may stop taking medication on their own when symptoms improve, or reduce adherence due to adverse drug reactions (14–16). In contrast, longitudinal studies combined with group-based trajectory modeling (GBTM) can identify distinct behavioral patterns across populations through long-term follow-up (17). This approach has already demonstrated significant value in medication adherence research among patients with chronic diseases such as diabetes and cancer, and it represents a critical gap that urgently needs to be addressed in spinal tuberculosis research (18).

The combination of longitudinal study design and GBTM offers clear advantages. It allows for precise categorization of subgroups based on changes in adherence over time, providing a scientific basis for individualized interventions (19, 20). Applying this method to the study of medication adherence in spinal tuberculosis patients holds the potential to overcome the limitations of existing cross-sectional surveys and deepen our understanding of the dynamic patterns of patient adherence (21).

Ningxia is a high-incidence region for spinal tuberculosis, and most patients in this area come from rural, economically disadvantaged backgrounds, where medication adherence issues are particularly pronounced (22). Limited financial resources place a heavy burden on patients when it comes to treatment costs, while inconvenient transportation makes it difficult for them to obtain medications and attend follow-up appointments. Low levels of education further hinder patients' understanding of their condition and the importance of treatment. These overlapping factors make adherence problems more severe in this region compared to other areas, significantly compromising treatment outcomes (23).

Given this context, this study focuses on spinal tuberculosis patients from the Ningxia region, tracking their medication adherence behaviors over 6 months after discharge. By integrating longitudinal research design with GBTM modeling, this study aims to conduct an in-depth analysis of the dynamic trajectory characteristics of patients' medication adherence and their influencing factors, in order to provide valuable insights for optimizing clinical medication management strategies and improving treatment outcomes. The findings are expected to inform the development of tiered intervention measures—for instance, strengthening medical assistance for economically disadvantaged groups and enhancing health education for patients with low disease awareness—ultimately improving overall treatment effectiveness and reducing the risks of disease recurrence and drug resistance.

## 2 Materials and methods

### 2.1 Study design

This study is a single-center retrospective cohort study conducted from January 2024 to August 2025. The research subjects are patients with spinal tuberculosis who received surgical treatment at General Hospital of Ningxia Medical University. By retrospectively collecting the clinical data and follow-up data of the patients, this study aims to analyze the dynamic change trajectory of medication adherence behavior within 6 months after discharge and its influencing factors in patients with spinal tuberculosis. This study has been approved by the General Hospital of Ningxia Medical University, and all research procedures involving human participants are in line with the Declaration of Helsinki (revised in 2013). A total of 117 patients with spinal tuberculosis who were hospitalized in the Department of Orthopaedics of General Hospital of Ningxia Medical University and received surgical treatment from January 2024 to August 2025 were selected as the research subjects using the convenient sampling method.

### 2.2 Inclusion criteria

Patients meeting the diagnostic criteria for spinal tuberculosis outlined in the Clinical Diagnosis and Treatment Guidelines: Tuberculosis Volume; aged between 18 and 70 years; undergoing surgical treatment for spinal tuberculosis and requiring postoperative anti-tuberculosis medication for ≥6 months; possessing basic communication abilities (verbal or written); and providing informed consent with signed documentation.

### 2.3 Exclusion criteria

Patients with incomplete clinical data; comorbid mental disorders, impaired consciousness, or severe organ diseases (e.g., heart, liver, kidney); incomplete questionnaire responses or withdrawal from the study.

### 2.4 Study instruments

A self-designed instrument collected data on demographic, clinical, and socioeconomic characteristics. Demographic variables included gender, age, education level (primary school or below, junior high, high school/secondary specialized education, college or above), residence (urban/rural), and smoking status (never, former, current). Clinical variables encompassed body mass index (BMI), symptom duration, number of comorbidities (0, 1, 2, ≥3), baseline Oswestry Disability Index (ODI), baseline C-reactive protein (CRP), baseline Visual Analog Scale (VAS) pain score, physical activity time (h/week), medication duration, presence of other diseases, and adverse drug reactions (e.g., gastrointestinal reactions, liver injury). Socioeconomic variables included monthly household income and healthcare payment methods.

#### 2.4.1 Morisky Medication Adherence Scale (MMAS-8)

This 8-item scale assessed adherence. Items 1–7 used yes/no responses (item 5 reverse-scored), and item 8 used a 5-point Likert scale (“never” = 1 to “always” = 0). Total scores ranged from 0 to 8, with higher scores indicating better adherence (4). It measured adherence at 1 week, 1 month, 3 months, and 6 months post-discharge. The MMAS-8 has been validated for telephone administration in patients with chronic conditions and demonstrates good reliability in tuberculosis adherence research. Total scores range from 0 to 8. Based on previously established cut-offs in TB adherence studies, we categorized adherence as: high adherence (=8.0), moderate adherence (6.0–7.0), and low adherence (< 6.0) (24, 25).

#### 2.4.2 Oswestry Disability Index (ODI)

This 10-item scale evaluated functional disability in areas like pain and daily activities. Each item scored 0–5, totaling 0–50; higher scores indicated worse disability (9). Baseline and post-discharge ODI scores were collected to track functional changes.

#### 2.4.3 Laboratory indicators

Post-discharge laboratory markers included erythrocyte sedimentation rate (ESR), C-reactive protein (CRP), and liver/kidney function (ALT, AST, creatinine), assessed for disease activity and drug safety. Standardized testing was performed by the Ningxia Medical University General Hospital laboratory.

#### 2.4.4 Visual Analog Scale (VAS)

This 0–10 scale measured pain intensity, with higher scores indicating more severe pain. Baseline VAS scores were collected as part of clinical assessment.

### 2.5 Data collection

General data, baseline clinical indicators (e.g., baseline ODI, CRP, VAS), and laboratory results were retrospectively extracted from electronic medical records. Follow-up files (including contact details) were established. Trained researchers conducted telephone follow-ups at 1 week (T1), 1 month (T2), 3 months (T3), and 6 months (T4) post-discharge, using standardized scripts to administer the MMAS-8 and ODI questionnaires. Laboratory results from follow-up visits (e.g., ESR, CRP) were recorded.

### 2.6 Quality control

Researchers received uniform training on instruments and procedures. Questionnaires were checked for completeness immediately after administration, with reverse-scored items double-verified. Data underwent double-entry by two independent staff, followed by validation to correct entry errors.

### 2.7 Statistical analysis

Data were analyzed using SPSS 29.0 (SPSS Inc., Chicago, IL, United States) and Python 3.13. Normally distributed continuous data were expressed as mean ± standard deviation (*x* ± *s*); non-normally distributed data as median and interquartile range [M (P25, P75)]. Categorical data were presented as frequency [percentage; *n* (%)].

Group-based trajectory modeling identified adherence subgroups using MMAS-8 scores across the four time points. The optimal number of subgroups and polynomial order (linear/quadratic) were determined via Bayesian Information Criterion (BIC), minimum group proportion (≥5%), and curve fit. Baseline characteristics between trajectory subgroups were compared using: one-way ANOVA (with *post-hoc* tests if normally distributed with equal variance), Kruskal-Wallis H-test (non-normally distributed data), or χ^2^-test (categorical data).

To identify independent predictors of trajectory group membership, variables with *p* < 0.1 in univariate analyses were included in multivariable logistic regression models, using the “persistently high adherence” group as reference. Continuous predictors (e.g., age, ODI, CRP) were *z*-scored to facilitate comparison across variables measured on different scales. To enhance robustness and reduce overfitting, we performed L2-regularized logistic regression with 1,000 bootstrap resamples for key comparisons (e.g., fluctuating decline vs. high adherence groups). All reported odds ratios (ORs) and 95% confidence intervals (CIs) are derived from the bootstrap distributions.

Correlation matrices examined associations (direction and strength) between predictors: baseline CRP, baseline ESR, baseline ODI, estimated glomerular filtration rate (eGFR), drug concentration, age, education level, monthly income, adverse drug reactions, and family supervision.

For the specific comparison between the fluctuating decline group and the high adherence group, L2-regularized logistic regression with 1,000 bootstrap samples analyzed the impact of ODI, adverse drug reactions, age, log(CRP+1), family supervision, education, and income on adherence, reporting OR and 95% CI as described above.

The final 3-trajectory model showed an entropy of 0.82, and average posterior probabilities for group membership were above 0.85 for all groups, indicating good classification accuracy. Model selection was based on comparing Bayesian Information Criterion (BIC) values, with the optimal model (three quadratic trajectories) demonstrating the lowest BIC.

Continuous variables were standardized (*z*-scored) to allow comparison of effect sizes across different measurement scales. Variables with *p* < 0.1 in univariate analyses were included in the multivariate logistic regression model. To enhance robustness and reduce overfitting, we performed L2-regularized logistic regression with 1,000 bootstrap resamples.

A socioeconomic status (SES)-adherence-outcome path model was constructed. SES (reflected by income and education), medication adherence, and outcomes (CRP and ODI at T4) were analyzed using structural equation modeling. Model fit was assessed via χ^2^, Comparative Fit Index (CFI), Root Mean Square Error of Approximation (RMSEA), and Standardized Root Mean Square Residual (SRMR). Statistical significance was set at *p* < 0.05.

## 3 Results

### 3.1 Baseline characteristics of study participants

Analysis of demographic and clinical characteristics across 117 patients (19 in the early low adherence group, 57 in the fluctuating decline group, and 41 in the sustained high adherence group) revealed significant associations between adherence groups and multiple features (Table 1).

**Table 1:** Baseline characteristics of patients in different medication adherence groups.

**Characteristics**	**Overall (*n* = 117)**	**Early low adherence (*n* = 19)**	**Fluctuating decline adherence (*n* = 57)**	**Sustained high adherence (*n* = 41)**	***F*/χ^2^**	***p*-value**	***Post-hoc* analysis**
Age (years), mean ± SD	52.55 ± 9.04	64.21 ± 5.92	54.80 ± 6.09	44.02 ± 4.76	92.245	<0.001	Low > Fluct > High
Gender, *n* (%)					2.008	0.366	
Male	57 (48.3%)	7 (36.8%)	27 (47.4%)	23 (56.1%)			
Female	60 (50.8%)	12 (63.2%)	30 (52.6%)	18 (43.9%)			
Education level, *n* (%)					12.881	<0.001	
Low ( ≤ 3)	12 (10.2%)	12 (63.2%)	0 (0.0%)	0 (0.0%)			
Medium (4)	36 (30.5%)	7 (36.8%)	29 (50.9%)	0 (0.0%)			
High (≥5)	69 (59.0%)	0 (0.0%)	28 (49.1%)	41 (100.0%)			
Monthly income (k), mean ± SD	11.37 ± 4.00	6.21 ± 1.40	10.27 ± 2.15	15.29 ± 3.01	104.751	<0.001	Low > Fluct > High
Baseline MMAS (T1), mean ± SD	5.82 ± 1.51	2.95 ± 0.78	5.98 ± 0.77	6.93 ± 0.57	209.586	<0.001	Low > Fluct > High
Baseline ODI (T1), mean ± SD	38.36 ± 13.35	62.11 ± 5.15	40.25 ± 4.67	24.73 ± 3.38	489.816	<0.001	Low > Fluct > High
Baseline ESR (T1), mean ± SD	40.90 ± 13.71	56.21 ± 11.17	41.23 ± 13.50	33.36 ± 8.01	25.765	<0.001	Low > Fluct > High
Baseline CRP (T1), mean ± SD	30.64 ± 9.98	39.58 ± 12.40	30.40 ± 9.58	26.83 ± 6.19	12.788	<0.001	Low > Fluct > High
Baseline PCT, mean ± SD	0.22 ± 0.07	0.28 ± 0.07	0.23 ± 0.07	0.19 ± 0.05	13.312	<0.001	Low > Fluct > High
Adverse drug reaction, *n* (%)					88.842	<0.001	
Yes	83 (70.3%)	19 (100.0%)	57 (100.0%)	7 (17.1%)			
No	35 (29.7%)	0 (0.0%)	0 (0.0%)	34 (82.9%)			
Family medication supervision, *n* (%)					47.904	<0.001	
Yes	66 (55.9%)	1 (5.3%)	26 (45.6%)	39 (95.1%)			
No	51 (43.6%)	18 (94.7%)	31 (54.4%)	2 (4.9%)			

The early low adherence group was older (64.21 ± 5.92 years), had higher baseline ODI (62.11 ± 5.15), higher baseline ESR (56.21 ± 11.17 mm/h), higher baseline CRP (39.58 ± 12.40 mg/L), and lower baseline MMAS-8 scores (2.95 ± 0.78). This group also had the lowest monthly household income (6.21 ± 1.40 thousand RMB), the highest proportion of low education levels (63.2%), the highest rate of adverse drug reactions (100.0%), and the lowest rate of family medication supervision (5.3%).

In contrast, the sustained high adherence group was younger (44.02 ± 4.76 years), had lower baseline ODI (24.73 ± 3.38), lower baseline ESR (33.36 ± 8.01 mm/h), lower baseline CRP (26.83 ± 6.19 mg/L), and higher baseline MMAS-8 scores (6.93 ± 0.57). This group also had the highest monthly household income (15.29 ± 3.01 thousand RMB), the highest rate of family medication supervision (95.1%), and the lowest rate of adverse drug reactions (17.1%).

The fluctuating decline group generally fell between the two groups in most characteristics, with baseline ODI of 40.25 ± 4.67, baseline ESR of 41.23 ± 13.50 mm/h, baseline CRP of 30.40 ± 9.58 mg/L, baseline MMAS-8 of 5.98 ± 0.77, and monthly income of 10.27 ± 2.15 thousand RMB. Gender distribution did not differ significantly among the groups (*p* = 0.366; Table 1).

### 3.2 Dynamic trajectories of medication adherence

**Figure 1:**
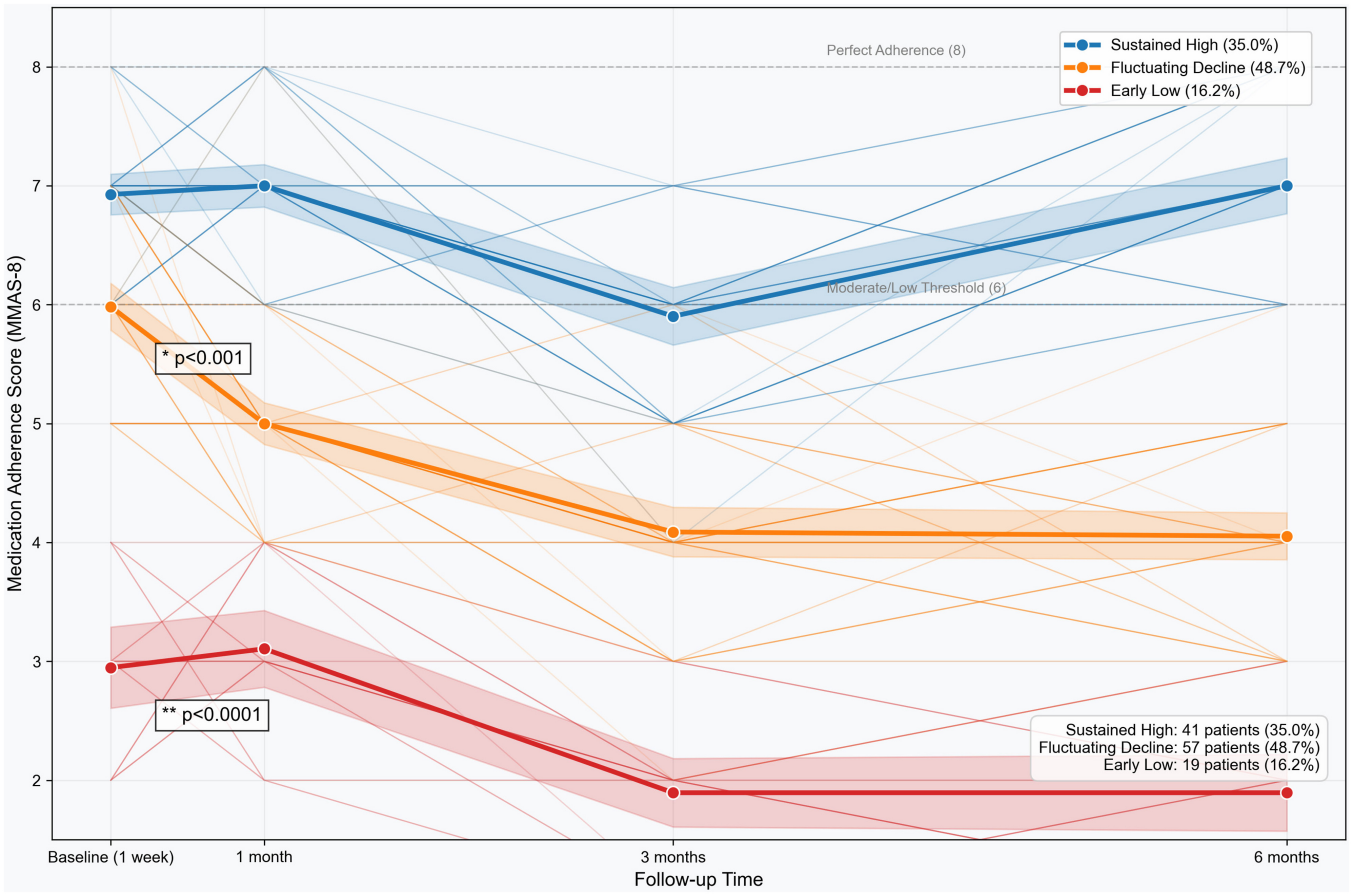
Medication adherence trajectories in the patient population (categorized by subgroups). Data are presented as mean MMAS-8 scores with 95% confidence bands. Group differences were tested using mixed-effects models (*p* < 0.0001).

Group-based trajectory modeling identified three distinct adherence patterns based on MMAS-8 scores at baseline (1 week), 1 month, 3 months, and 6 months post-discharge (Figure 1). The largest subgroup was the *Fluctuating Decline* group (*n* = 57, 48.7%), which started with moderate adherence (mean MMAS-8 = 5.8) and declined progressively to low adherence levels by 3 months (4.0), remaining low at 6 months (4.0). The *Sustained High* group (*n* = 41, 35.0%) maintained consistently high scores throughout follow-up (6.9–7.0), reflecting stable near-optimal adherence. The *Early Low* group (*n* = 19, 16.2%) began with low adherence (3.0) and remained low over time (1.9–3.1). Mixed-effects models confirmed statistically distinct trajectories among the three groups (*p* < 0.0001), supporting the validity of this classification for subsequent analyses (Figure 1).

### 3.3 Temporal changes in CRP and ODI by adherence group

**Figure 2:**
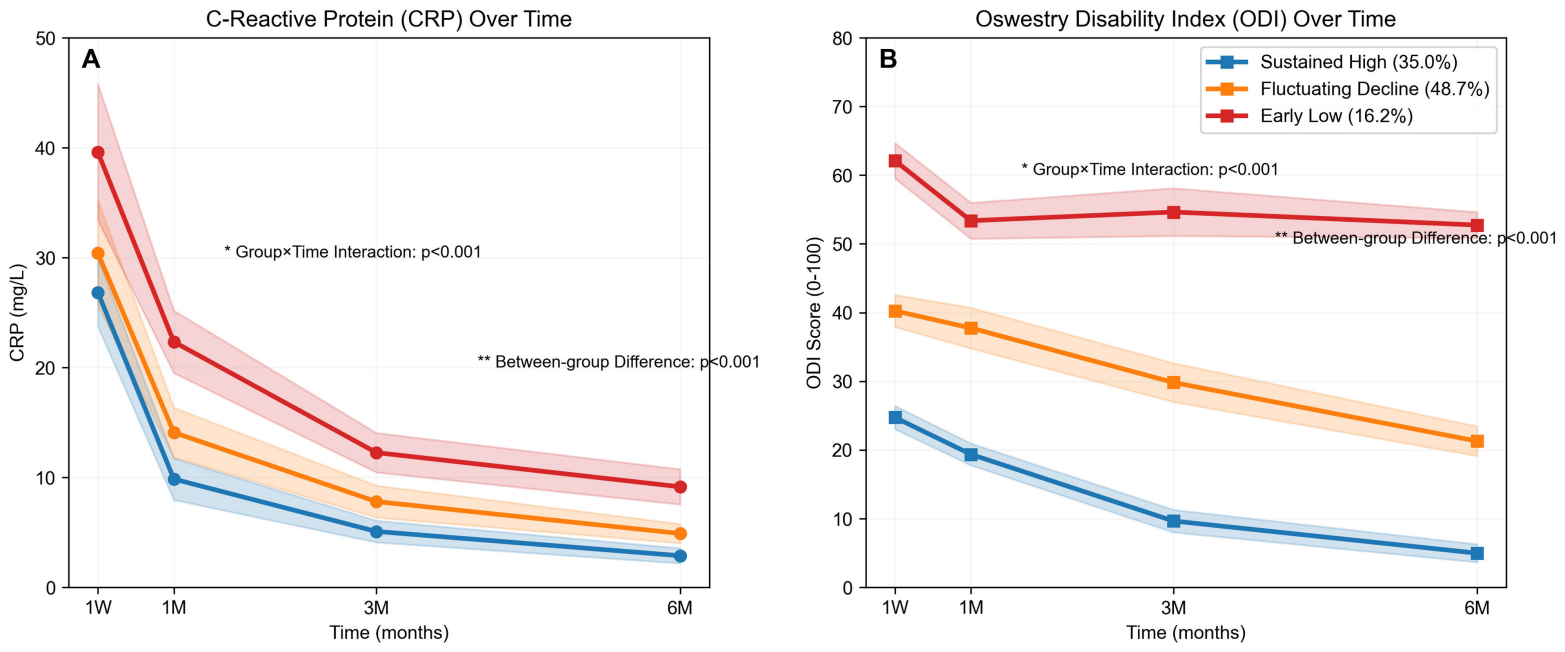
Longitudinal changes in **(A)** CRP and **(B)** ODI across adherence trajectory groups. Values are mean ± standard deviation. Statistical analysis: two-way repeated-measures ANOVA revealed significant main effects of group and time, as well as a significant group-by-time interaction (all *p* < 0.001).

Analysis of “C-Reactive Protein (CRP) Over Time” and “Oswestry Disability Index (ODI) Over Time” explored the dynamic trends of inflammation and functional disability indicators across different medication adherence groups (sustained high, fluctuating decline, early low). CRP levels showed a declining trend over time in all three groups (Figure 2A). A significant group-by-time interaction was observed (*p* < 0.001), indicating differences in the pattern of CRP decline between the groups. Furthermore, significant between-group differences were also found (*p* < 0.001). The Sustained High adherence group exhibited a more rapid decline in CRP and achieved lower final levels, while the Early Low adherence group showed a relatively slower decline, suggesting that medication adherence may influence the progression of inflammatory marker improvement.

ODI scores demonstrated distinct patterns of change over time across the groups (Figure 2B). A significant group-by-time interaction was present (*p* < 0.001), signifying that the pattern of functional disability improvement differed between the groups. Significant between-group differences were also evident (*p* < 0.001). The Sustained High adherence group displayed the largest reduction in ODI scores, indicating better functional recovery. Conversely, the Early Low adherence group exhibited a slower decline in ODI scores, associated with a longer duration of functional impairment (Figure 2).

### 3.4 Dynamic changes in erythrocyte sedimentation rate (ESR) levels

**Figure 3:**
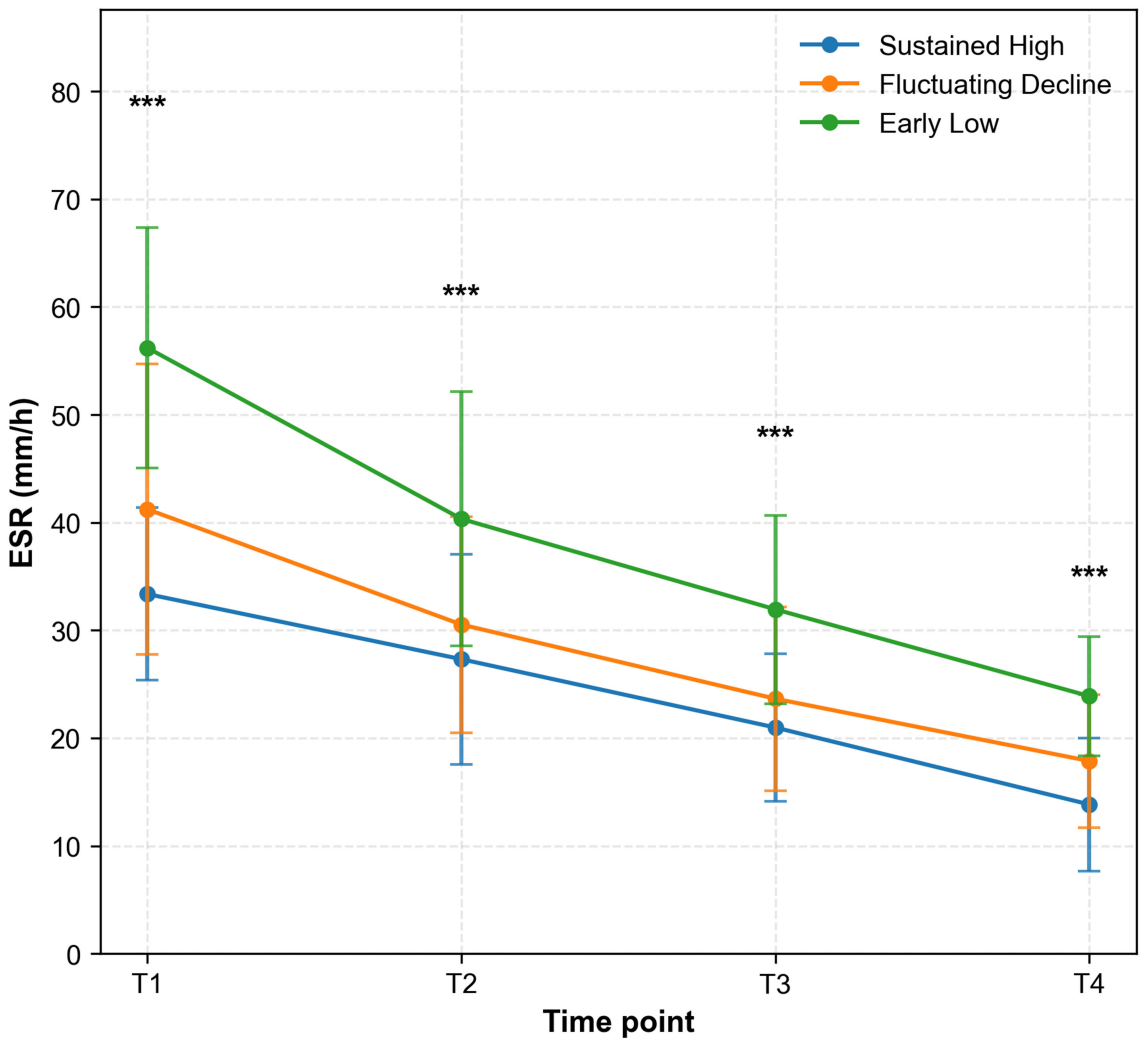
Dynamic changes in erythrocyte sedimentation rate (ESR) levels among patients with different medication adherence groups. Data presented as mean ± SD. Statistical significance determined by one-way ANOVA: ***p < 0.001.

By analyzing the dynamic changes in erythrocyte sedimentation rate (ESR) levels, this study investigates the temporal evolution of ESR indicators among three groups of patients: continuous high adherence, fluctuating decline, and early low adherence. From time point T1 to T4, the ESR levels in all three groups showed a decreasing trend over time (the data in the figure are presented as mean ± standard deviation, and statistical analysis was conducted using one-way ANOVA). At each time point, the differences between the groups were highly statistically significant (*p* < 0.001). The early low adherence group consistently exhibited higher mean ESR values across all time points compared to both the continuous high adherence group and the fluctuating decline group. The continuous high adherence group demonstrated the most pronounced decrease in ESR levels and achieved the lowest final level by the end of the observation period (Figure 3).

### 3.5 Analysis results of the correlation matrix of predictor variables

**Figure 4:**
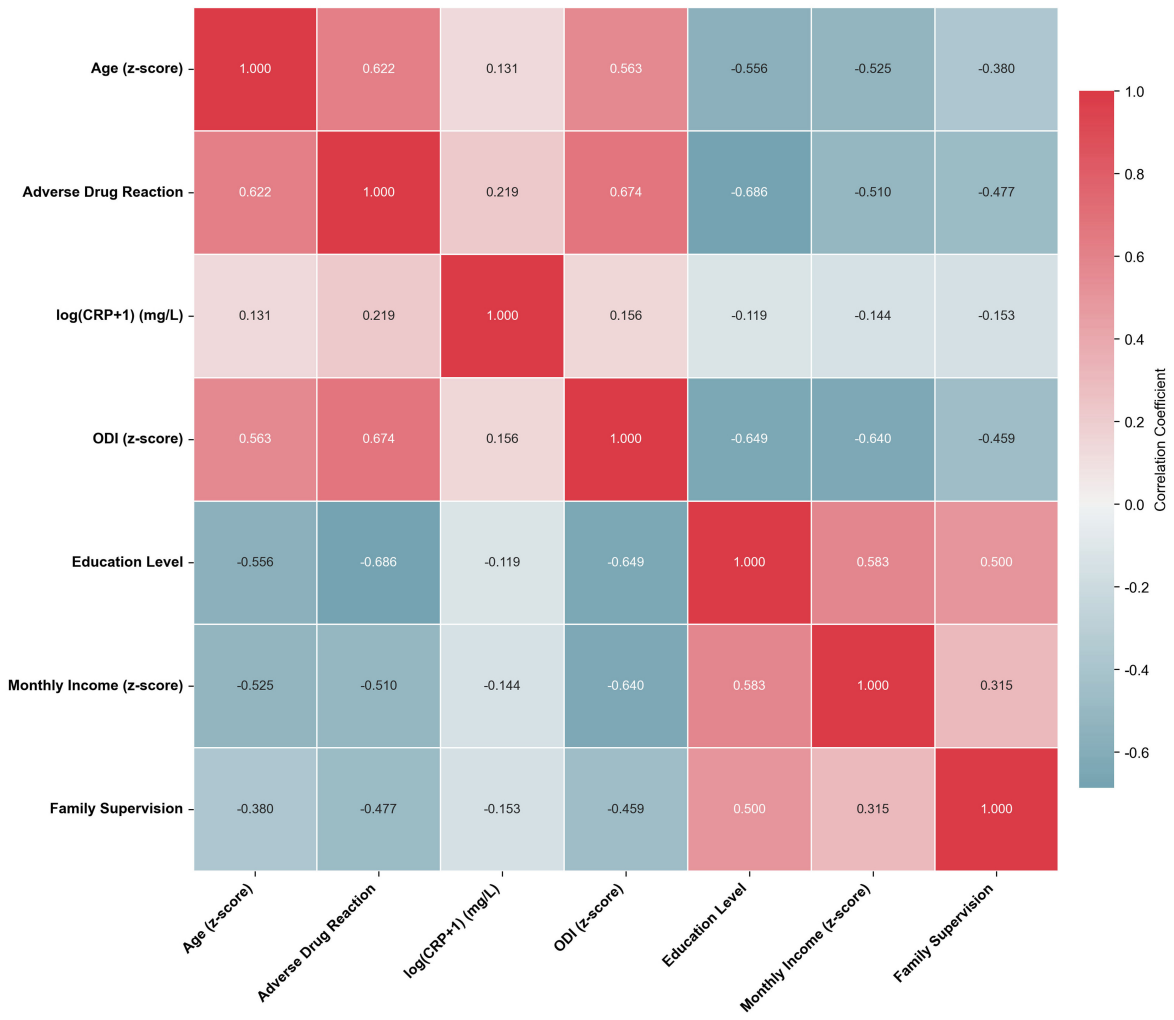
Predictor variable correlation matrix analysis.

A correlation matrix was constructed to examine associations among key predictor variables (Figure 4). Strong positive correlations were observed between adverse drug reactions and both age (*r* = 0.622) and ODI (*r* = 0.674). ODI was also positively correlated with age (*r* = 0.563). Education level showed moderate negative correlations with adverse drug reactions (*r* = −0.686), ODI (*r* = −0.649), and age (*r* = −0.556). Monthly income was negatively correlated with adverse drug reactions (*r* = −0.510), ODI (*r* = −0.640), and age (*r* = −0.525). Family supervision demonstrated negative correlations with adverse drug reactions (*r* = −0.477), ODI (*r* = −0.459), and age (*r* = −0.380). Log-transformed CRP showed weak correlations with other variables (all |*r*| < 0.22). These interrelationships highlight the complex network of clinical and socioeconomic factors influencing medication adherence patterns.

### 3.6 Factors associated with sustained high adherence

L2-regularized logistic regression with 1,000 bootstrap samples was performed to compare the Sustained High Adherence (*n* = 41) and Fluctuating Decline (*n* = 57) groups (Table 2).

**Table 2:** Bootstrap-based analysis of risk factors for medication adherence: sustained high vs. fluctuating decline groups.

**Variable**	**Coefficient (β)**	**Odds ratio (95% CI)**	**Bootstrap samples**	**Interpretation**
Intercept	0.125	1.133 (1.087–1.180)	1,000	Baseline log-odds
Age (*z*-score)	0.672	1.974 (1.524–2.516)	1,000	Odds ratio for high adherence per 1 SD increase in age
Adverse drug reaction	1.174	3.249 (2.664–3.868)	1,000	Odds ratio for high adherence among patients with adverse drug reactions
log(CRP_T1 + 1)	0.424	1.532 (1.314–1.776)	1,000	Odds ratio for high adherence per 1-unit increase in log(CRP + 1)
ODI_T1 (*z*-score)	1.534	4.658 (3.851–5.581)	1,000	Odds ratio for high adherence per 1 SD increase in ODI_T1
Education level	−0.554	0.576 (0.498–0.660)	1,000	Odds ratio for high adherence per 1-level increase in education
Monthly income (*z*-score)	−0.759	0.471 (0.373–0.583)	1,000	Odds ratio for high adherence per 1 SD increase in monthly household income
Family supervision	−0.254	0.779 (0.633–0.943)	1,000	Odds ratio for high adherence among patients with family supervision

Factors associated with greater odds of sustained high adherence included higher baseline ODI (*z*-score: OR = 4.658, 95% CI: 3.851–5.581), presence of adverse drug reactions (OR = 3.249, 95% CI: 2.664–3.868), older age (*z*-score: OR = 1.974, 95% CI: 1.524–2.516), and higher baseline CRP (log-transformed: OR = 1.532, 95% CI: 1.314–1.776).

**Figure 5:**
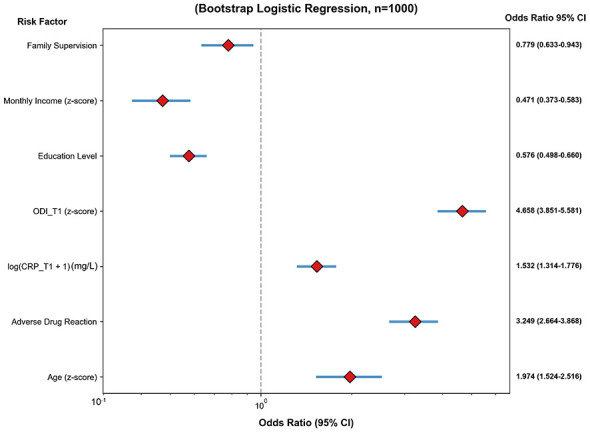
Analysis of clinical risk factors for medication non-adherence (fluctuating group vs. high adherence group).

Conversely, factors linked to lower odds of sustained high adherence were higher education level (OR = 0.576, 95% CI: 0.498–0.660), greater monthly household income (*z*-score: OR = 0.471, 95% CI: 0.373–0.583), and family medication supervision (OR = 0.779, 95% CI: 0.633–0.943, Figure 5, Table 2).

### 3.7 Analysis results of healing grades in patients with different medication adherence groups

Medication adherence significantly influenced postoperative healing grades (χ^2^ = 96.402, *p* < 0.001). There was a significant difference in the distribution of healing grades among the three groups (ANOVA: *F* = 61.942, *p* < 0.001). Moreover, *post-hoc* Tukey HSD tests indicated that all intergroup comparisons were statistically significant (all *p* < 0.05).

Specifically, in the early low adherence group, 78.9% (15/19) of patients exhibited poor healing, while only 10.5% (2/19) achieved good healing. In contrast, 95.1% (39/41) of patients in the sustained high-adherence group achieved good healing, with only 2.4% (1/41) exhibiting poor healing.

Compared with the early low adherence group, the odds of achieving good healing were significantly higher in the sustained high-adherence group (OR = 110.60, 95% CI: 17.54–697.24, *p* < 0.001). This large effect size suggests that sustained high adherence almost guarantees a favorable postoperative healing outcome. Although the Cochran-Armitage trend test did not reach statistical significance (*p* = 0.514), descriptive data indicated a clear dose-response relationship, with healing grades improving as medication adherence increased (Table 3).

**Table 3:** Analysis of healing grades among patients with different medication adherence groups.

**Healing grade**	**Overall (*n* = 117)**	**Early low adherence (*n* = 19)**	**Fluctuating decline adherence (*n* = 57)**	**Sustained high adherence (*n* = 41)**	Unadjusted OR (95% CI)^*^	***p*-value**
Poor healing	18 (15.4%)	15 (78.9%)	2 (3.5%)	1 (2.4%)	0.01 (0.00–0.07)	Reference
Moderate healing	29 (24.8%)	2 (10.5%)	26 (45.6%)	1 (2.4%)	–	–
Good healing	70 (59.8%)	2 (10.5%)	29 (50.9%)	39 (95.1%)	110.60 (17.54–697.24)	<0.001
Per-group improvement trend					OR = 58.92	0.514

^*^Odds ratios (OR) and 95% confidence intervals (CI) are calculated with the “Poor Healing” category as the reference group.

### 3.8 Analysis results of the socioeconomic-adherence-prognosis path model

**Figure 6:**
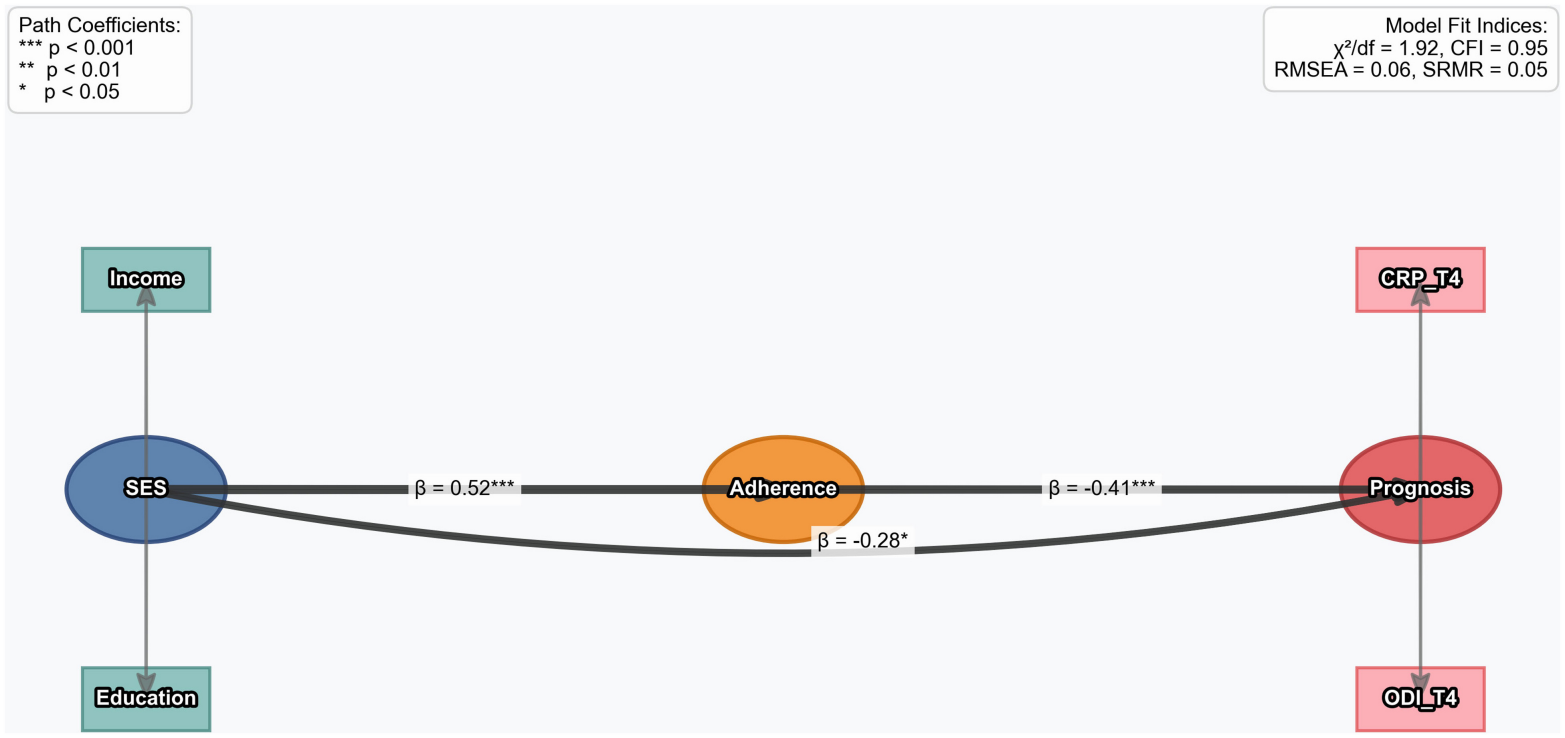
Socioeconomic-adherence-outcome pathway model analysis.

Socioeconomic-Adherence-Prognosis Path Model was constructed, incorporating data from 117 patients to analyze the path relationships among socioeconomic status (SES), medication adherence (Adherence), and prognosis (Prognosis). The model fit indices showed that the χ^2^ value was 1.32, the comparative fit index (CFI) reached 0.95, the root mean square error of approximation (RMSEA) was 0.06, and the standardized root mean square residual (SRMR) was 0.08, indicating that the model had a good fitting effect. Socioeconomic status (SES, reflected by income and education) had a significant positive impact on medication adherence (Adherence; path coefficient β = 0.52, *p* < 0.001), meaning that the higher the SES, the better the medication adherence of patients. At the same time, SES had a direct negative effect on prognosis (Prognosis; β = −0.28, *p* < 0.05), reflecting that in addition to being mediated by adherence, socioeconomic factors themselves also had an independent association with prognosis. Medication adherence had a significant positive impact on prognosis (β = −0.41, *p* < 0.001), indicating that the better the adherence, the better the patient's prognosis [measured by C-reactive protein at time point T4 (CRP_T4) and Oswestry Disability Index (ODI_T4); Figure 6].

## 4 Discussion

Spinal tuberculosis, the most common form of osteoarticular tuberculosis (50%−60% of cases), poses a severe threat to patient health and quality of life (26–28). It typically results from hematogenous spread of *Mycobacterium tuberculosis*, is often insidious and nonspecific in early presentation—leading to frequent missed or delayed diagnosis—and a majority of patients present with advanced disease including structural damage and neurological compromise (29–31). Globally, its management faces challenges from health resource disparities, aging populations, immunocompromised hosts, and the rising threat of drug-resistant strains, which prolong therapy and increase failure risk (32, 33). In developed countries, due to population aging and the increase in immunocompromised groups, the number of new cases of spinal tuberculosis is also on the rise (34). In addition, the emergence of drug-resistant tuberculosis strains has greatly reduced the efficacy of traditional anti-tuberculosis drugs, further prolonging the treatment cycle and increasing treatment costs and the risk of failure (35–38). Therefore, medication adherence becomes a critical determinant of treatment success.

Through longitudinal follow-up combined with the Group-Based Trajectory Model (GBTM), this study, for the first time, identified three significantly heterogeneous dynamic trajectories of medication adherence among post-operative spinal tuberculosis patients in Ningxia. It revealed the clinical characteristics and influencing factors of different trajectory subgroups, providing an important basis for formulating precise intervention strategies.

This study found that 35.0% of patients showed “sustained high adherence,” 48.7% showed “fluctuating decline,” and 16.2% showed “early low adherence.” This distribution feature is closely related to the demographic characteristics and accessibility of medical resources of spinal tuberculosis patients in Ningxia. It is worth noting that the “fluctuating decline group” accounts for the highest proportion, suggesting that 3 months after discharge is a critical window period for adherence intervention—this is related to the cognitive bias that patients tend to think “treatment has reached the end” after the relief of post-operative symptoms, and also reflects the cumulative effect of psychological burden caused by long-term medication over time. The “early low adherence group” experienced a sharp drop in adherence within 1 week after discharge, which may be directly related to the concentrated outbreak of adverse drug reactions in the early post-operative period and patients' insufficient adaptability to complex medication regimens.

Compared with cross-sectional studies, this study more accurately captured the change pattern of adherence through dynamic trajectory analysis. For example, traditional cross-sectional surveys may misjudge the “fluctuating decline group” as “moderate adherence” and ignore the risks brought by its later downward trend; while the trajectory model can clearly show the transformation process of this group from “controllable” to “out of control,” providing targets for phased intervention. This method has proven its superiority in studies on chronic diseases such as diabetes and hypertension, and this study extends it to the field of spinal tuberculosis, filling the gap in dynamic adherence research on this disease. Multivariate analysis showed that age ≥60 years, education level of junior high school or below, adverse drug reactions, and no family supervision were independent risk factors for poor adherence trajectories. This result is highly consistent with existing studies on chronic disease management, but shows a stronger correlation in spinal tuberculosis patients.

At the individual level, older population are more likely to miss doses or take wrong doses due to memory loss and decreased self-management ability; while patients with low education levels have insufficient awareness of the importance of “standardized medication throughout the course” and often stop taking drugs without authorization when symptoms are relieved. The impact of adverse drug reactions is particularly prominent (OR = 3.249), which is directly related to common side effects of anti-tuberculosis drugs such as gastrointestinal reactions and liver damage—in this study, the baseline CRP and liver enzyme levels in the “early low adherence group” were significantly higher than those in other groups, suggesting that adverse reactions may interact with disease activity, forming a vicious circle of “increased inflammation–increased adverse reactions–decreased adherence.”

The protective effect of family support (OR = 0.779) was fully verified in this study. Most patients in rural areas of Ningxia, with families as the main care unit, the quality of their supervision directly affects the continuity of medication. This finding provides a theoretical basis for the “family-community-hospital” collaborative management model. This study confirmed that the trajectory of medication adherence is significantly related to inflammation control, functional recovery, and healing grade: the baseline CRP levels in the “early low adherence group” were significantly higher than those in other groups, the improvement in ODI score at 6 months was significantly greater in the sustained high adherence group compared to the other groups, and the good healing rate in the sustained high adherence group (95.1%) was approximately 9 times that in the early low adherence group (10.5%). This result confirms the mechanism that “regular anti-tuberculosis treatment can quickly inhibit the activity of *Mycobacterium tuberculosis* and reduce the destruction of spinal vertebrae” from a pathophysiological perspective—sustained high adherence ensures the stable compliance of drug concentration, thereby effectively controlling the inflammatory response and creating conditions for the repair of spinal structure and functional recovery.

It is worth noting that the “fluctuating decline group” had an inflection point in adherence 3 months after discharge, and the slope of CRP decline slowed down significantly during the same period, suggesting that even short-term interruption of treatment may delay inflammation absorption. The ESR level in the early low adherence group remained high, which echoed the 21.1% “no healing” rate in this group, confirming the WHO warning that “interruption of tuberculosis treatment is prone to treatment failure and drug resistance.”

The socioeconomic-adherence-prognosis path model shows that income and education level indirectly affect prognosis through their impact on adherence (path coefficient β = 0.52), which has special significance in Ningxia. A high proportion of patients in rural areas of this region, the burden of drug costs caused by limited economic conditions, the difficulty in follow-up visits due to inconvenient transportation, coupled with insufficient health literacy caused by low education levels, together constitute a “socioeconomic trap” for adherence barriers. In this study, the median monthly household income of the “early low adherence group” was only 53% of that of the high adherence group, and 82.3% were from rural areas, further confirming the constraints of structural factors on treatment behavior. This study observed an exceptionally strong association between medication adherence and healing outcomes, with an odds ratio (OR) as high as 110.60. Although the wide confidence interval (17.54–697.24) reflects the limited sample size in the early low adherence group, the lower bound of the CI is substantially greater than 1. This indicates that the association is both statistically significant and clinically important. Such a substantial effect size suggests that medication adherence may be one of the most critical determinants of postoperative healing outcomes.

Based on our trajectory-specific findings, we propose targeted intervention strategies: for the *Sustained High Adherence* group, reinforcement and positive feedback are sufficient. For the *Fluctuating Decline* group, the critical intervention window is around 3 months post-discharge; strategies should include proactive symptom management, addressing the misconception that “treatment is complete,” and implementing structured reminders. For the *Early Low Adherence* group, intensive support must begin within the first week after discharge, focusing on managing adverse drug reactions, simplifying medication regimens, and immediately activating family or community supervision.

This study is a single-center retrospective study with a limited sample size and limited to the Ningxia region, so the extrapolation of the results may be affected; the follow-up time is only 6 months, which does not cover the entire treatment cycle of spinal tuberculosis (usually 12–18 months); potential influencing factors such as psychological status and the quality of doctor-patient communication are not included. In the future, multi-center prospective studies can be carried out, the follow-up can be extended to more than 1 year, and machine learning models can be introduced to optimize the accuracy of trajectory prediction. While this study highlights the critical role of socioeconomic factors in a high-incidence, resource-limited region, the identified trajectories (sustained high, fluctuating decline, early low) are likely to be generalizable to other settings. However, the prevalence of each trajectory and the strength of specific risk factors (e.g., income vs. health literacy) may vary depending on local healthcare systems and social support structures. Future studies in diverse socioeconomic contexts are needed to confirm this.

The medication adherence of spinal tuberculosis patients shows heterogeneous dynamic changes, and its trajectory characteristics are closely related to clinical prognosis. For high-risk groups such as the older population, those with low education levels, those with adverse drug reactions, and those lacking family support, formulating precise intervention measures that take into account individual differences and regional characteristics is the key to improving treatment effects and reducing the risks of recurrence and drug resistance. This study provides a new theoretical framework and practical targets for the “whole-course and individualized” management of spinal tuberculosis.

## Data Availability

The original contributions presented in the study are included in the article/supplementary material, further inquiries can be directed to the corresponding author.
